# Numerical and Experimental Investigation on the Surface Defect Generation during the Hot Extrusion of Al6063 Alloy

**DOI:** 10.3390/ma14226768

**Published:** 2021-11-10

**Authors:** Namsu Park, Yeonghwan Song, Seon-Ho Jung, Junghan Song, Jongsup Lee, Heejong Lee, Hyun-Min Sung, Gihyun Bae

**Affiliations:** 1 Molding & Metal Forming R&D Department, Korea Institute of Industrial Technology, Incheon 21999, Korea; jsh5626@kitech.re.kr (S.-H.J.); jhsong@kitech.re.kr (J.S.); jongsup@kitech.re.kr (J.L.); 2 Functional Materials and Components R&D Group, Korea Institute of Industrial Technology, 137-41, Gwahakdanji-ro, Sacheon-myeon, Gangneung-si 25440, Korea; yhsong0105@kitech.re.kr; 3 Department of Mechanical Engineering, Inha University, 100, Inha-ro, Michuhol-gu, Incheon 22212, Korea; 4 LG Electronics, Production Engineering Research Institute, 222, LG-ro, Jinwi-myeon, Pyeongtaek-si 17709, Korea; heejong2.lee@lge.com (H.L.); hyunmin.sung@lge.com (H.-M.S.)

**Keywords:** extrusion, surface defect, white line, metallurgical analysis, thermomechanical FE simulation

## Abstract

The surface quality control of extruded products is a critical concern in the home appliance manufacturing industry owing to the increasing need for products with a high surface quality, in addition to the essential mechanical properties of the final product. The underlying issue with achieving high-quality extrusion products is that surface defects, especially those resulting in surface gloss differences, called white line defects, are only observed after surface treatment. In this study, we aim to investigate the cause of white line defect generation on the surface of an extruded product. Accordingly, an experimental extrusion program is established using an L-shaped die that has a noticeable change in its bearing length along the inner corner of its cross-sectional profile. Laboratory-scale experiments were performed for the L-shaped extrusion of homogenized Al 6063 alloy at various ram speeds, in order to induce surface defects, considering the production yield rate required for mass production. Subsequently, the microstructural changes near the surface failure region were investigated using an arbitrary Lagrangian–Eulerian (ALE) technique-based thermomechanical finite element (FE) analysis. To scale-up the defect observation method from laboratory-scale to production-scale manufacturing and confirm the reproducibility of the surface defect, scaled-up L-shaped extrusions were performed in an actual industrial production line. Finally, the potential cause of white line defect generation is discussed by comparing the numerical and metallurgical analyses, including the scanning electron microscopy (SEM) and electron backscatter diffraction (EBSD) observations.

## 1. Introduction

Various types of open or closed shape profiles can be formed by aluminum extrusion, using a porthole die with numerous holes for the billet to pass through. The dimensions of each porthole can vary in accordance with the target product shape, which determines the overall volumetric flow rate of the metal in each cavity of the die. The metal flow rate at the outlet must be carefully controlled based on the die bearing length to prevent the twisting, bending, tearing, and formation of waves in the extruded profile. These geometrical defects also mutually depend on process parameters such as the ram speed and the initial temperature of the die and container, which can further influence the surface quality of the product after metal finishing processes such as heat treatment and anodizing. Consequently, a significant amount of research has been conducted over the last few decades, primarily based on experimental investigations, to determine the best extrusion conditions, such as the production yield rate, surface quality, and product strength, to satisfy industrial requirements, and to systematically analyze the mechanism of defect formation during extrusion based on numerical and metallurgical observations. 

To standardize the die design process, Miles et al. [1] discussed the requirements for selecting the bearing length, based on common principles and techniques used by skilled die designers. Lesniak and Libura [2] investigated the influence of pocket die geometry with varying thicknesses on the metal flow and surface quality of the extrudate, compared to that of conventional flat dies. Sheppard [3] conducted a microstructural investigation that revealed the ram speed and temperature play a vital role in the subsequent heat treatment and surface quality of the metal product. He also confirmed that the breakthrough pressure can be accurately predicted if the prevailing Zener‒Hollomon parameter is defined accurately. Clade and Sheppard [4] investigated the high temperature flow stress-, surface-, and microstructure-related extrusion limits of AA 6063, based on experimental data. The pressure requirements, microstructural features, and occurrence of surface defects were characterized under laboratory conditions using mathematical formulas, within the limit diagram framework that contains the microstructural and topological information. Donati and Tomesani [5] conducted an extensive investigation to determine the correlation between the die design and the extrusion process of AA 6082 considering a broad range of operating conditions, and assessed the workability area without tearing defects. 

Numerous researchers have also investigated the various defect modes that occur over the life cycle of the die and concluded that defect generation is strongly related to process parameters such as the die design and heat treatment conditions used to manufacture a product. Arif et al. [6] investigated the die failure mechanism during extrusion based on numerous observations of die failures involving different die profiles. To prevent overheating between the die and the workpiece material, Hölker et al. [7] proposed an extrusion die design with integrated local cooling, based on the additive manufacturing technology of selective laser melting. They reported that internal die cooling near the die bearings can prevent surface defects such as a rough surface even at high ram speeds, owing to a significant reduction in the exit temperature of the sample. Jhavar et al. [8] observed that die failures under operating conditions are primarily due to high thermal shock, cyclic loading, and corrosion, and owing to faulty design, defective material, mishandling, and force majeure under accidental conditions. Qamar et al. [9] discussed the sources of major die defects, preventive measures, and die correction operations, based on a frequency-based statistical analysis of the defect data from an actual medium-to-large size commercial extrusion plant. Owing to several experimental and metallurgical investigations on dies and operating conditions and their relation to product quality, as well as the experiential expertise of industry experts, the acceptable range of the initial conditions for various process parameters, including billet preparation, have been established to achieve the cost-effective manufacturing of extrusion products, using various kinds of aluminum alloys. The operating parameters for extrusion typically include the preheat temperatures of the billet, die, and container, the billet length and ram speed, and the cooling period and aging conditions of the product, based on the aluminum alloy used.

Numerical techniques for extrusion analysis under severe plastic deformations have also been developed to determine the cause of various defects and provide suitable countermeasures to prevent extrusion flaws, based on a comprehensive understanding of the physical mechanism and the variations in the state variables, such as strain, stress, and temperature, as the metal flows through the die orifice, which cannot be measured experimentally [10]. The updated Lagrangian (UL) method is commonly used in conjunction with various remeshing techniques to study severe plastic deformations that cause undesired distortions in the finite elements. This numerical approach has a strong effect on the direct evaluation of the transient behavior of the material flow in the cavity of the die during the extrusion process. Lee et al. [11] studied uniform microstructure generation based on a die profile optimization method that employs a thermomechanical finite element (FE) model, considering the microstructure evolution that occurs during the hot extrusion process. Zhou et al. [12] simulated the entire cycle of aluminum extrusion through transient and steady states using the DEFORM 3D software, based on the UL approach. Numerical observations of physical quantities such as the velocity, equivalent strain, and billet temperature revealed that the extrusion process occurs under non-steady-state conditions even in the steady-state, due to continuous heat generation and sticking conditions between the billet and the container interface. Li et al. [13] investigated the formation mechanism of transverse welds, considering the metal flow mechanism in aluminum extrusion, to provide general guidance. Donati and Tomesani [14] presented a general methodology to predict the weld quality in extruded aluminum profiles by evaluating the pressure, flow stress, and velocity fields on the welding surface through numerical analyses. 

Observations of the extrusion process, based on the UL approach, have provided a detailed understanding of its physical mechanism, especially considering the changes in the state variables under non-steady-state conditions. However, the remeshing technique used in the UL approach to prevent the high level of element distortion, which is usually observed in the region where the cross-section changes rapidly, significantly increases the total number of FE meshes. This leads to a drastic increase in the computation time considering the increased degrees of freedom and size of the data record space [15,16]. For cases involving abrupt changes in the mass flow rate due to complicated die cavity shapes, the UL approach typically encounters mesh degeneration problems that affect the numerical accuracy of extrusion analyses. Consequently, the arbitrary Lagrangian‒Eulerian (ALE) method has also received significant attention from researchers. One of the most valuable advantages of the ALE method is that element distortion and entanglement are eliminated, as the Eulerian characteristic allows the FE mesh to exist independent of the material flow. In general, the ALE method consists of two steps, corresponding to the updated Lagrangian and Eulerian processes, respectively: in the UL process, the numerical analysis is carried out at each time step until the required convergence condition is attained; in the Eulerian process, each nodal point is rearranged, generating a new mesh system with the same topology for the deformed domain, which differs from the traditional remeshing technique. Consequently, compared to the conventional UL approach, the ALE method can significantly reduce the computational effort required to analyze engineering problems with large deformations. Yang et al. [17] developed rigid-viscoplastic FE programs based on the ALE description to investigate the effects of various design parameters, including die lands and porthole dies, in the hot extrusion process. Lof and Blokhuis [18] employed an equivalent bearing model assuming an isothermal condition for the temperature cycle of the extrusion process to simulate complex extrusion dies for practical applications and generate rough estimates of the extrusion pressure and exit velocity. Zhang et al. [19] numerically observed the effect of the extrusion stem speed on the metal flow behavior associated with the distribution of temperature, extrusion force, and welding pressure, and determined the optimum stem speed for flow velocity distribution.

Studies have shown that a reliable set of extrusion conditions, including the design of the die and portholes, can be determined through parametric FE analyses and the experiential expertise of skilled industry experts, to design cost- and time-effective extrusion processes. As the extrusion industry now has an in-depth understanding of the metal flow in the die, simple geometric defects, such as a bent extrudate profile, are effectively managed in a timely manner, and are no longer considered to be a significant problem. Owing to the increasing need for extruded products with a high surface quality, especially for home appliance products, surface quality control has become the primary concern in the industry today, besides satisfying the basic mechanical requirements of the final product. The surface quality of a product relies not only on the relative stress and strain variations in the product, but also on the surface treatment conditions that affect the thermomechanical states associated with the changes in the microstructure during the hot extrusion process [20,21]. Although significant attempts have been made in recent decades to establish an industrial guide to address the various types of surface failures in extruded products, a comprehensive understanding of defect mechanisms, especially of white line generation on the surface of the final product after anodizing treatment, is still lacking. Some researchers have presented metallurgical clues for white line generation based on microscopic and macroscopic investigations on the surface quality of the extruded products during the surface treatment process [22]. These studies are a meaningful source of information in the industry. However, in practice, without a concurrent understanding of the mechanical influences of stress, strain, and temperature distribution that are strongly related to the product profile, the usefulness of these studies is limited. As the mechanical state of an extruded product is affected by the interactions between the physical and metallurgical conditions present during the production process [23,24], the white line defect can even occur under a safe process window of manufacturing the target profile, based on the variations in the die and lip design, even for the same exit profile. 

In this study, we attempted to determine the mechanism of white line generation based on a comprehensive investigation of the mechanical and metallurgical properties of the extrudate, through numerical and microstructural analyses, including SEM and EBSD observations, of the location of the surface failure. To induce the white line surface defect, we first performed laboratory-scale experiments involving the extrusion of an L-shaped sample that has a noticeable change in its bearing length along the inner corner of its profile cross-section at various ram speeds. The hardening and deformation behaviors of the billet were then investigated by performing an FE analysis under the ALE description, and ascertaining the relationship between the variations in the state variables and the instant of the actual surface defect generation. To confirm that the description of the deformation mechanism provided by the laboratory-scale test is applicable to the industry, a scaled-up version of the L-shaped extrusion was manufactured in an actual industrial production line, considering the ram speed required for a practical production yield rate. Finally, the mechanism of the formation of microscopic surface irregularities was analyzed considering both the physical and metallurgical aspects of white line defect generation, based on the investigation of the surface roughness at the failure site and the influence of the strain and friction concentration due to a rapid increase in temperature at the failure site.

## 2. Materials and Methods

**Figure 1:**
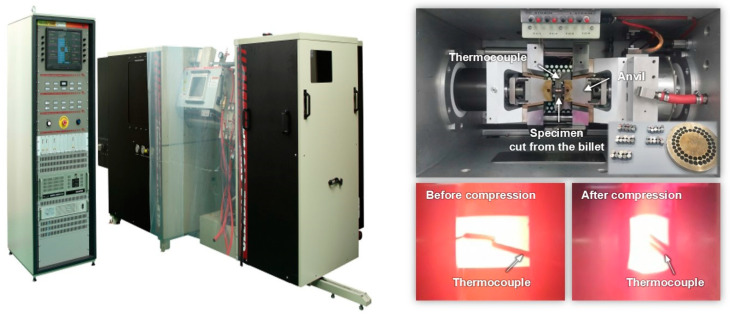
Gleeble 3500 thermomechanical simulator and cylindrical specimens fabricated from the billet.

A hot compression test was performed using the Gleeble 3500 (Dynamic Systems Inc., Austin, TX, USA) thermomechanical simulator shown in Figure 1 to evaluate the strain rate and temperature-dependent strain hardening of homogenized and extruded 6063 aluminum alloy.

To reliably assess the plastic deformation, a cylindrical specimen with dimensions of Ø10 × 12 mm was prepared from the same billet used in the actual extrusion process. The experimental program consisted of sixteen different combinations of the target strain rate (0.005, 0.05, 0.5, and 5 s^−1^) and temperature (250, 350, 450, and 550 °C), considering the changes in stress and strain under the harsh environmental conditions experienced by the billet during the extrusion process. Each compression test was performed thrice to confirm the reproducibility of the material hardening behavior under a given test condition. 

### 2.1. Experiment

To reliably evaluate the material properties of the metal, variations in the target conditions during the test must be avoided, as the mechanical response, including the material ductility, corresponding to a certain strain level is likely to be affected by changes in physical quantities such as the material anisotropy, strain rate, and temperature [25,26,27]. Considering the large deformation of the billet at a high strain rate and temperature, the mechanical characterization was performed using the Gleeble 3500 thermomechanical simulator that features a feedback control system that allows the target strain rate and temperature to be appropriately controlled in the gauge region during the plastic deformation of the specimen. The initial target temperature was attained through direct resistance heating and maintained at a constant value. The compression test was then performed using the stroke control of the anvil to achieve the target strain rate. 

**Figure 2:**
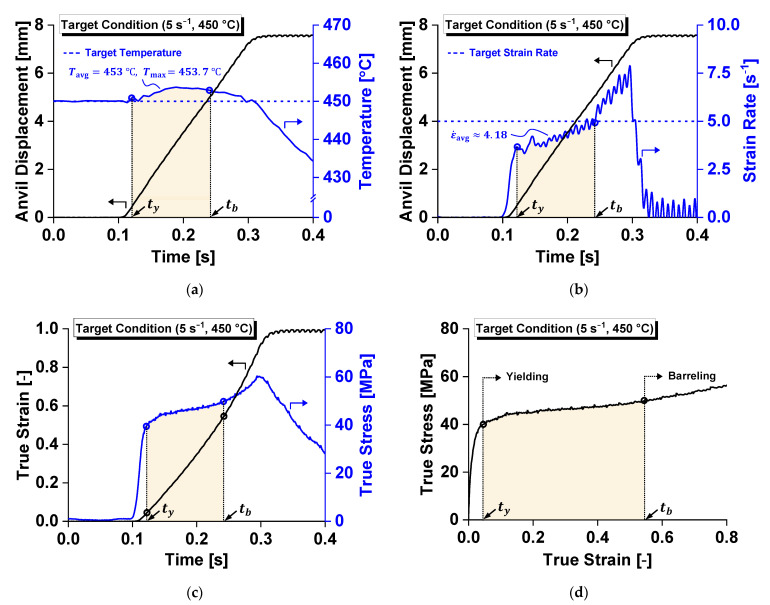
Variations in the physical quantities during compression under the target test conditions of a strain rate of 5 s^−1^ and a temperature of 450 °C: (**a**) temperature; (**b**) strain rate; (**c**) in situ stress and strain responses; and (**d**) flow stress curve.

Figure 2 illustrates the variations in the strain, strain rate, temperature, and stress–strain relationship at a target strain rate of 5 s^−1^ and temperature of 450 °C, which were satisfactorily controlled during the compression test. Some differences between the target and evaluated strain rates are unavoidable owing to the difficulties associated with instantaneous feedback control considering the high stroke rate of the moving anvil. 

Under this test condition, the temperature increased by 4 °C and the average strain rate was 4.2 s^−1^. When the nominal strain reached 0.53, the cylindrical specimen started to undergo barreling, resulting in a non-uniform stress state away from uniaxial compression. Consequently, to reliably evaluate the mechanical response, the relationship between the true stress and true strain was characterized until the point at which the barreling began. This point can be numerically determined as it is characterized by a rapid change in the instantaneous strain rate, with increased plastic deformation, as indicated in Figure 2b. Table 1 summarizes the strain rate, temperature, and nominal strain at the instant of barreling and yield stress, together with their standard deviations relative to each target test condition. The representative values of strain rate and temperature were computed as a time-integral average of each state variable over the duration from the initial yielding, $t_{y}$, to the barreling of the cylindrical specimen, $t_{b}$: $X_{\mathrm{avg}}=\int_{t_{y}}^{t_{b}}X\;\mathrm{dt}/\left(t_{b}-t_{y}\right)$, where X substitutes for $\dot{\varepsilon }$ and T. 

**Figure 3:**
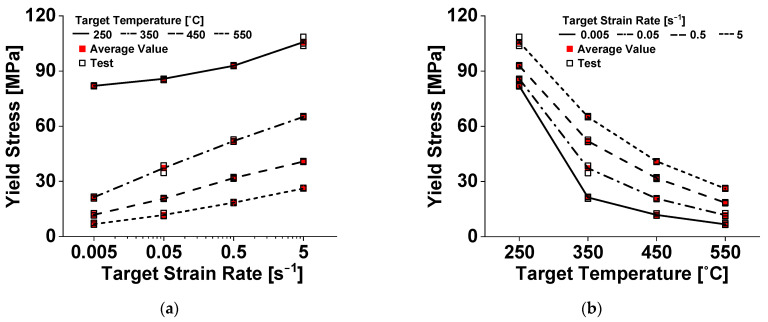
Compressive yield stress of Al 6063 alloy dependent on: (**a**) strain rate; and (**b**) temperature.

The experimental observation confirmed that Al 6063 alloy has both positive strain rate dependence and negative temperature dependence on the flow stress level, and exhibits lesser sensitivity to the strain rate during material yielding compared to the temperature, as shown in Figure 3. 

At temperatures of 250–350 °C, the decrease in the flow stress level is relatively high, which can be attributed to dynamic softening due to the coarsening of precipitates during the hot deformation of a precipitation-hardening aluminum alloy [28]. The material characterization revealed that the target strain rate and temperature conditions cannot be perfectly maintained and vary with the extent of plastic deformation. Therefore, the evolution of these physical quantities must be carefully considered, especially while calibrating the parameters of the strain rate- and temperature-dependent strain-hardening law, to reliably describe the material deformation. 

### 2.2. Constitutive Modeling

An Arrhenius-type constitutive equation modified by Lin et al. [29] was used to predict the flow stress of Al 6063 alloy herein, considering the effects of the strain rate and temperature on hardening, which were described using the Zener‒Hollomon parameter of Z as an exponential equation:

(1) $$\dot{\varepsilon }=AF\left(\sigma \right)\exp\left(-\frac{Q}{RT}\right),$$

with

(2) $$F\left(\sigma \right)\equiv \{\begin{array}{cc} \sigma^{n} & \alpha \sigma <0.8\\ \exp\left(\beta \sigma \right) & \alpha \sigma >1.2\\ {\left[\sinh\left(\alpha \sigma \right)\right]}^{n} & \mathrm{for}\;\mathrm{all}\;\sigma \end{array},$$

(3) $$Z=\dot{\varepsilon }\exp\left(\frac{Q}{RT}\right),$$

where $\dot{\varepsilon }$, Q, R, and *T* denote the strain rate, activation energy, universal gas constant of 8.314 J∙mol^−1^∙K^−1^, and absolute temperature in K, respectively. A and n are material constants. α and Z, respectively, denote stress multiplier defined as β/m and changes in temperature or strain rate to the strain hardening response. Considering the strain hardening at low (ασ<0.8) and high (ασ<1.2) stress levels, the power and exponential laws can be defined as:

(4) $$\dot{\varepsilon }=B\sigma^{m\left(\varepsilon ,T\right)},$$

(5) $$\dot{\varepsilon }=C\exp\left[\beta \left(\varepsilon ,T\right)\sigma \right].$$

From the logarithmic viewpoint of Equations (4) and (5), the linear relationship between strain, strain rate, and stress can be expressed as:

(6) $$\ln\left(\sigma \right)=\frac{1}{m\left(\varepsilon ,T\right)}\ln\left(\dot{\varepsilon }\right)-\frac{1}{m\left(\varepsilon ,T\right)}\ln B,$$

(7) $$\sigma =\frac{1}{\beta \left(\varepsilon ,T\right)}\ln\left(\dot{\varepsilon }\right)-\frac{1}{\beta \left(\varepsilon ,T\right)}\ln C.$$

If the slopes 1/m(ε,T) and 1/β(ε,T) do not depend significantly on the temperature for a certain strain $\varepsilon =\varepsilon^{*}$, the values of the slopes can be linearly averaged as $1/{\bar{m}|}_{\varepsilon =\varepsilon^{*}}$ and $1/{\bar{\beta }|}_{\varepsilon =\varepsilon^{*}}$, with respect to the temperature. Accordingly, the exponent of $\bar{\alpha }$ for the strain $\varepsilon^{*}$ can be determined as $\bar{\alpha }=\bar{\beta }/{\bar{m}|}_{\varepsilon^{*}}$. From Equation (2), the activation energy Q and the exponent n can be expressed for all stress levels, at the strain $\varepsilon =\varepsilon^{*}$ as:

(8) $$Q={Rn\frac{d\left\{\ln\left[\sinh\left(\alpha \sigma \right)\right]\right\}}{d\left(1/T\right)}|}_{\varepsilon =\varepsilon^{*}},$$

(9) $$\ln\left[\sinh\left(\alpha \sigma \right)\right]=\frac{\ln\dot{\varepsilon }}{n}+\frac{Q}{nRT}-\frac{\ln A}{n}.$$

Mathematically, Q and 1/n represent the slopes in the two-dimensional domains of ln[sinh(ασ)]−1/T and $\ln\left[\sin h\left(\alpha \sigma \right)\right]-\ln\dot{\varepsilon }$. Accordingly, these terms can also be defined in terms of the linearly averaged values of each slope, ${\bar{Q}|}_{\varepsilon =\varepsilon^{*}}$ and $1/{\bar{n}|}_{\varepsilon =\varepsilon^{*}}$, considering the strain rate and temperature, respectively. Consequently, A can be evaluated using Equation (9), i.e., ${\bar{A}|}_{\varepsilon =\varepsilon^{*}}=\dot{\varepsilon }/{\left[\sinh\left({\bar{\alpha }|}_{\varepsilon =\varepsilon^{*}}\sigma \right)\right]}^{{\bar{n}|}_{\varepsilon =\varepsilon^{*}}}\exp\left(-\frac{{\bar{Q}|}_{\varepsilon =\varepsilon^{*}}}{RT}\right)$, considering the strain rate and temperature at a strain $\varepsilon^{*}$. To accurately describe the stress response with plastic deformation in the given test conditions, each parameter of $\overset{-}{x}$ was evaluated at a strain interval of 0.05 and fitted using the 5th-order polynomial function in terms of the true strain: $x=x\left(\varepsilon \right)=\sum_{i=0}^{5}c_{i}x^{i}$, where *x* substitutes for α, n, Q, and lnA, respectively. The coefficients of each function are summarized in Table 2. The variations in the strain rate and temperature were further considered to study the evolution of each parameter with plastic deformation. 

Combining the Arrhenius-type equation and the Zener‒Hollomon parameter given by Equations (1)–(3), the flow stress response can be expressed in an explicit form that includes the state variables of the strain, strain rate, and temperature:

(10) $$\sigma =\sigma \left(\varepsilon ,\dot{\varepsilon },T\right)=\frac{1}{\alpha }\ln\left\{{\left(\frac{Z}{A}\right)}^{1/n}+{\left[{\left(\frac{Z}{A}\right)}^{2/n}+1\right]}^{1/2}\right\}.$$

**Figure 4:**
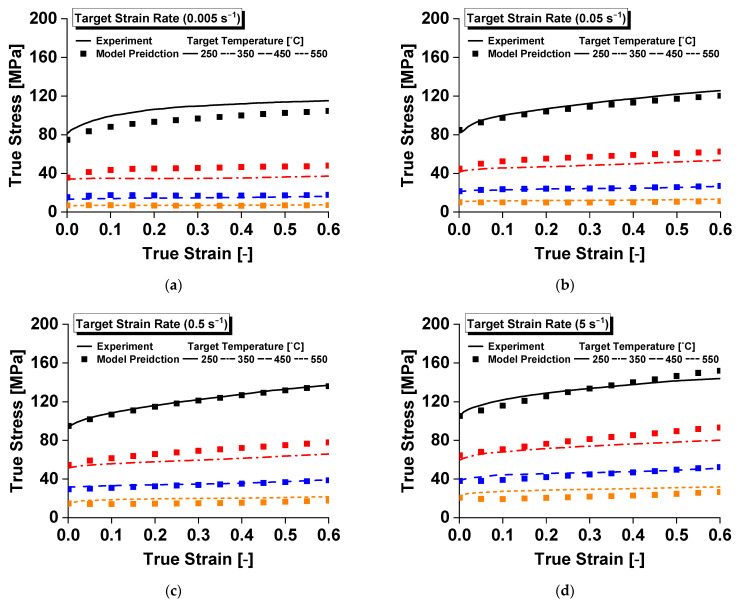
Temperature-dependent compressive yield stress of Al 6063 alloy at target strain rates of: (**a**) 0.005 s^−1^; (**b**) 0.05 s^−1^; (**c**) 0.5 s^−1^; and (**d**) 5 s^−1^.

To evaluate the performance of the model in predicting the flow stress responses in Figure 4, the Pearson correlation coefficient, r, was evaluated.

(11) $$r=\frac{\sum^{\;}\left(\sigma_{i}^{e}-{\bar{\sigma }}^{e}\right)\left(\sigma_{i}^{p}-{\bar{\sigma }}^{p}\right)}{\sqrt{\sum^{\;}{\left(\sigma_{i}^{e}-{\bar{\sigma }}^{e}\right)}^{2}\sum^{\;}{\left(\sigma_{i}^{p}-{\bar{\sigma }}^{p}\right)}^{2}}}\in \left[-1,\;1\right],$$

with

(12) $$\bar{\sigma }=\frac{1}{n_{d}}\sum_{i=1}^{n_{d}}x_{i},$$

where the superscripts e and p denote the experimental and model prediction values, respectively, and $n_{d}$ is the total number of data points. Herein, *r* exhibited a linear correlation between the experimental and the predicted values over the entire range of the strain, strain rate, and temperature: 0 for no linear correlation, and ±1 for perfectly positive and negative linear correlations, respectively. The evaluated value of r between the experimental and predicted values was 0.979, which indicates that the proposed model can describe the flow stress response of Al 6063 in the overall range of test conditions herein with reasonable accuracy. 

**Figure 5:**
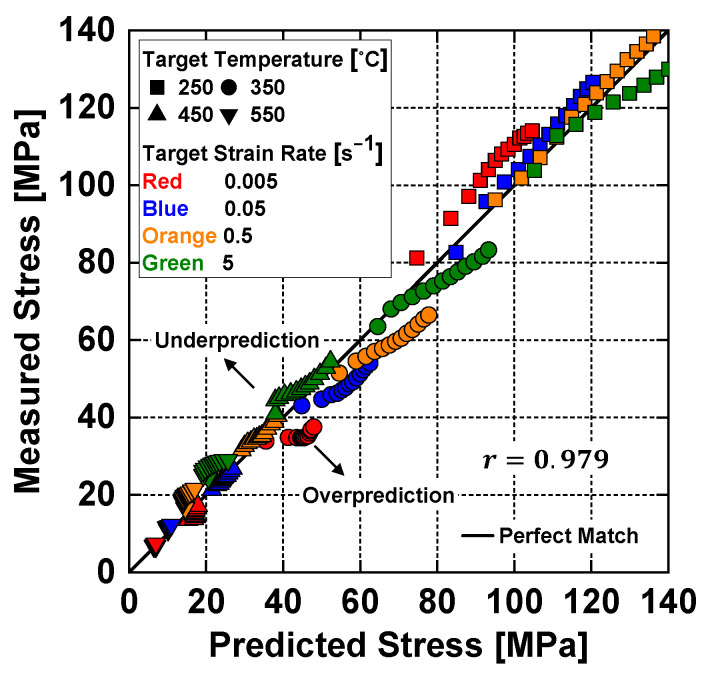
Scatter plot of the Pearson correlation coefficient between the experimental and numerical prediction values obtained from the strain rate- and temperature-dependent strain hardening model.

A scatter plot of the Pearson correlation coefficient is presented in Figure 5.

## 3. Laboratory Scale L-Shaped Sample Extrusion

### 3.1. Finite Element Modeling

**Figure 6:**
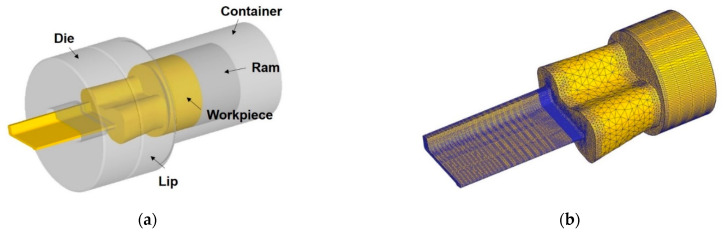
Modeling of the L-shaped sample extrusion for a numerical analysis based on the arbitrary Lagrangian‒Eulerian method: (**a**) schematic illustration; and (**b**) finite element discretization of the workpiece.

**Figure 7:**
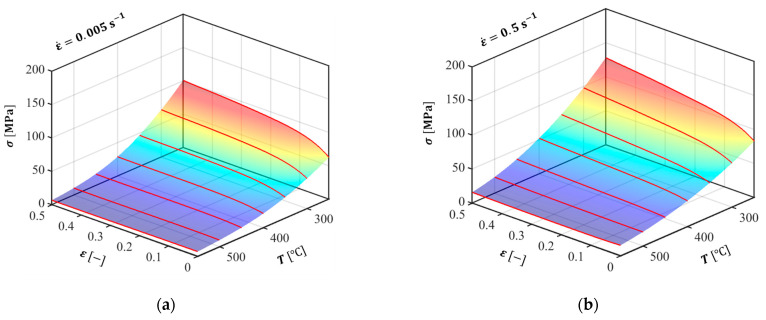

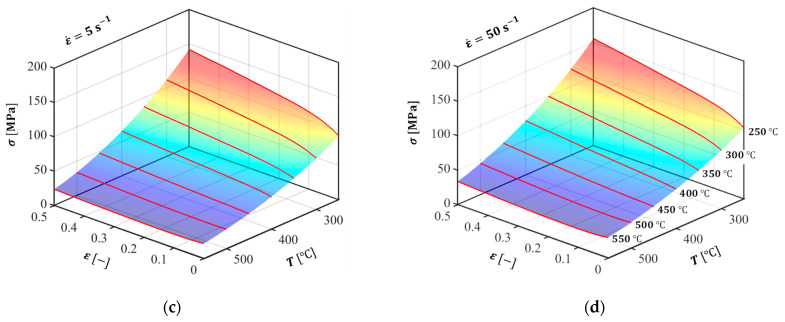
Flow stress responses predicted by the Arrhenius-type strain rate- and temperature-dependent strain hardening model: (**a**) 0.005 s^−1^; (**b**) 0.5 s^−1^; (**c**) 5 s^−1^; and (**d**) 50 s^−1^. The red lines represent the flow stress curves used in the FE analysis.

For the FE analysis of the L-shaped sample extrusion process, the Al 6063 alloy workpiece was modeled as a rigid-plastic finite element considering constitutive behavior of the flow stress in accordance with the strain, strain rate, and temperature, as discussed in Section 2.2. The other parts of the die, lip, container, and ram were described as rigid bodies, as shown in Figure 6a. Various strain rate and temperature conditions were considered in the FE simulation by assigning the piecewise linear data, represented by the solid red lines in Figure 7, as plastic properties. A detailed numerical formulation of the ALE description is provided in Appendix A.

The elastic region was described by assuming isotropic linear elasticity for the Al 6063 alloy (E=68.9 GPa, ν=0.33). Notably, in the ALE analysis using DEFORM 3D (Scientific Forming Technologies Corporation, Ohio, USA), the numerical computation required that the workpiece model completely filled the inner cavities of the die and container and filled the extruded part along the extrusion direction to some extent. 

Figure 6b represents the discretization of the workpiece with a controlled mesh size, especially for the bearing and its regions, considering the accuracy and computational efficiency of the numerical analysis. The total number of elements and nodes generated were 247,284 and 50,330, respectively. The boundary conditions of the extrusion analysis were established, as listed in Table 3, considering the operating processes of aluminum extrusion in the industry. Thermal properties from the DEFORM library were applied to describe the heat transfer between the contact pairs.

**Figure 8:**
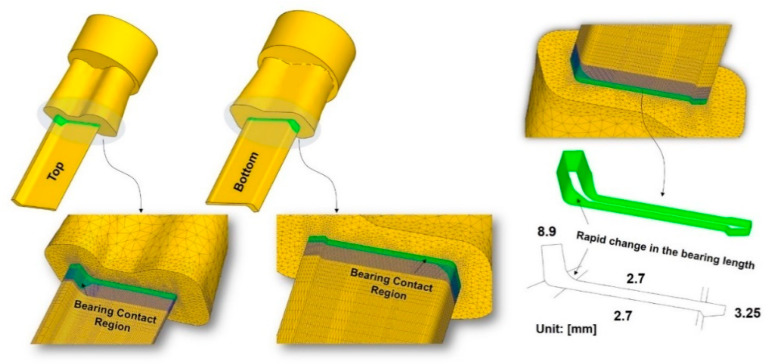
Bearing location and dimensions of the L-shaped sample extrusion.

The FE simulation was conducted at various ram speeds (3.5, 6, 9, and 12 mm∙s^−1^) to investigate the mechanical response and its influence on the surface quality of the extruded workpiece, particularly in the region with rapid changes in the bearing length, as shown in Figure 8. The average exit velocity at each ram speed was calculated based on the cross-sectional ratio of the inlet and the outlet, assuming constant volume flow. A ram speed of 9 mm∙s^−1^ almost corresponds to the exact level of the volume velocity required to achieve the production yield rate of the L-shaped sample considered herein, which varies based on the sample shape and size, along with additional product requirements such as the surface quality, and product strength.

### 3.2. Numerical Analysis Results

**Figure 9:**
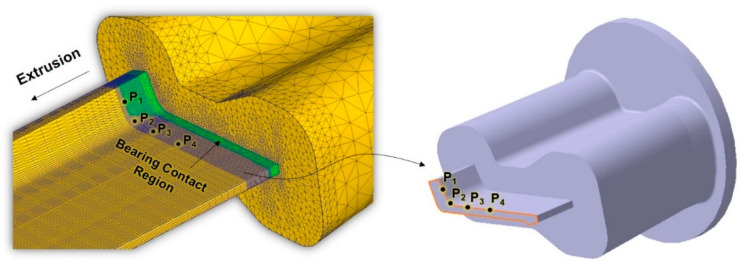
Locations used to evaluate the mechanical responses with the variation in ram speed.

To investigate the influence of the ram speed on the mechanical response, four material points were manually selected along the edge of bearing, as shown in Figure 9. For the numerical evaluation of the mechanical response of the workpiece under steady-state conditions, the cross-section that includes the selected material points is located at the top of the bearing, particularly at the instant when all the contacts between the die and workpiece over the bearing area are detached. 

**Figure 10:**
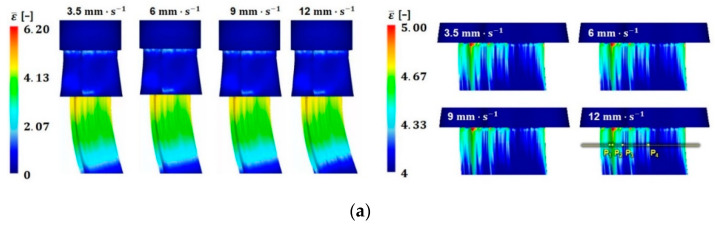

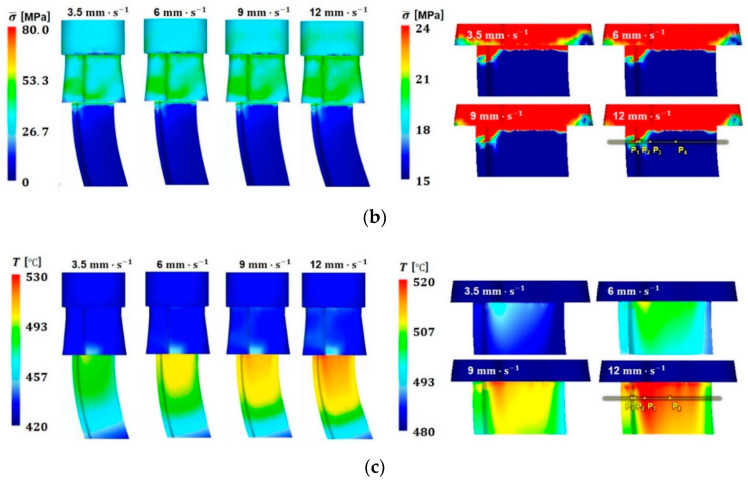
Distribution of the physical quantities with ram speed: (**a**) equivalent plastic strain; (**b**) von Mises effective stress; and (**c**) temperature.

Figure 10 presents the distribution of the equivalent plastic strain, von Mises effective stress, and temperature, together with their magnified views. The results of the FE analysis indicate that severe plastic deformation is localized in the region where the bearing length changes rapidly. In addition, the temperature at the localized strain region also increases with the increase in ram speed. 

**Figure 11:**
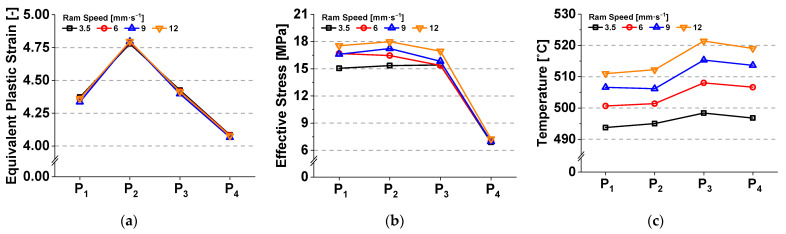
Influence of ram speed on the variations in physical quantities: (**a**) equivalent plastic strain; (**b**) von Mises effective stress; and (**c**) temperature.

Comparing the results at each material point, the localized stress and temperature increase with the increase in ram speed. However, the variations in the equivalent plastic strain are almost negligible as the ram speed increases, as shown in Figure 11.

**Figure 12:**
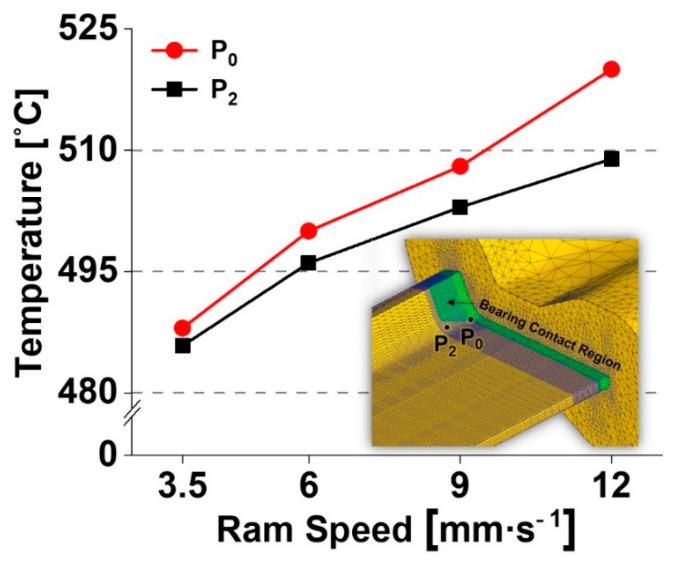
Temperature localization at the inner corner on the top surface of the L-shaped sample.

This implies that the extent of material deformation is likely associated with the final geometric shape of the workpiece, including the bearing length and the form of the internal cavities of the die, under the given extrusion conditions. Meanwhile, the increase in the localized temperature between P_2_ and P_3_ indicates a relatively strong dependence on ram speed compared to the strain and stress. Furthermore, the high level of temperature localization can be rapidly initiated, particularly at P_0_, where the bearing length starts to change significantly, as shown in Figure 12. This observation is possibly due to the additional frictional heat that is primarily generated by the increase in the relative velocity between the contact region of the bearing and the workpiece, and is relevant to the volumetric metal flow at the bearing outlet.

### 3.3. Experimental Analysis of the Defect Region

**Figure 13:**
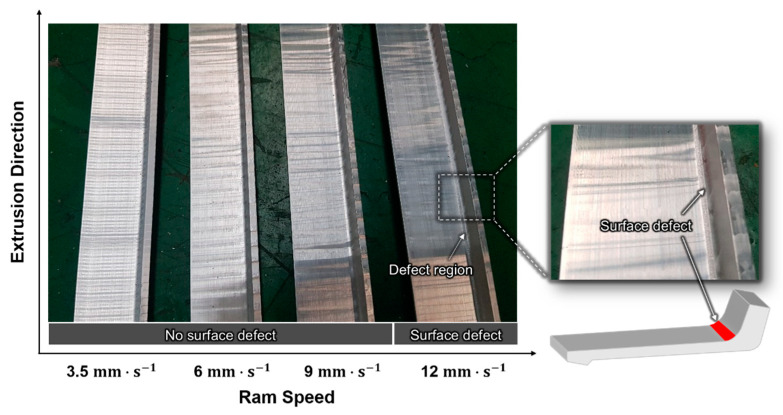
L-shaped samples extruded at various ram speeds.

In conjunction with the numerical analysis of aluminum extrusion, an experimental analysis of the L-shaped sample extrusion was performed using an extrusion press to investigate the effect of the mass flow variation, especially at the location where the bearing length changes rapidly. The test apparatuses included a container and a heating furnace for the die, lip, and billet. The die and lip were made of SKD61 steel and subjected to nitriding several times to harden the surface of the tool. The experimental conditions were the same as those used in the FE analysis. Figure 13 shows the variation in the experimental results with ram speed. The streak-line defect was detected along the region of interest only at a ram speed of 12 mm∙s^−1^; it was not detected at the other ram speeds. 

**Figure 14:**
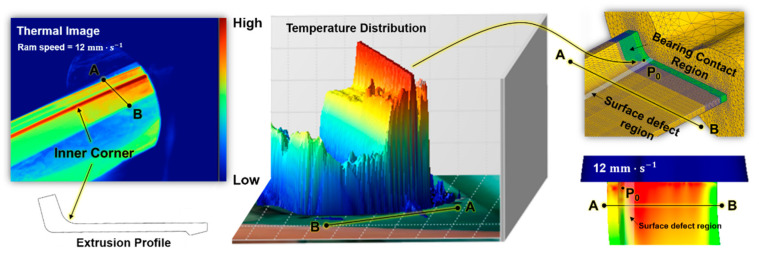
Temperature distribution measured using a Fluke Ti400 infrared camera.

To investigate the temperature distribution along the workpiece during the extrusion process, thermal images of the workpiece were recorded using a Fluke Ti400 infrared camera (Fluke Corporation, Washington, DC, USA), with a thermal emissivity, ϵ = 0.25, as shown in Figure 14. 

**Figure 15:**
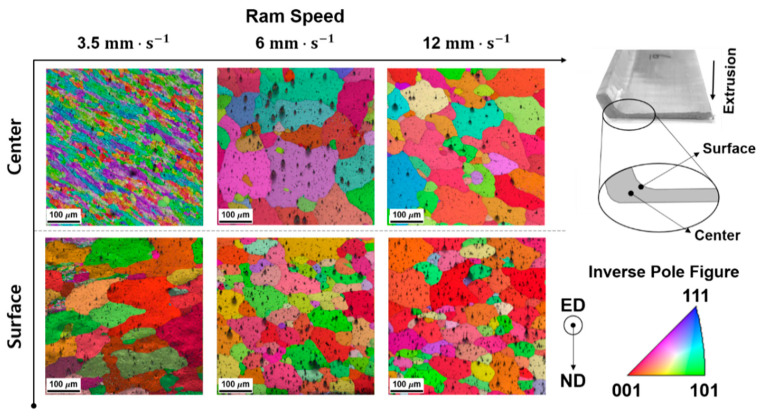
EBSD observations for the samples tested at various ram speeds.

Considering the numerical simulation results, the temperature concentration was experimentally observed along the region where the surface defect occurred. However, the absolute temperature recorded during the experiment could not be directly compared to the FE prediction as the thermal image only represents the relative temperature distribution, based on the thermal emissivity value. In addition to the macroscopic observation of the extrusion quality, metallurgical analyses were also performed at the surface defect sites, by measuring electron backscatter diffraction (EBSD) maps in plane parallel to the extrusion direction. It is noted that samples were prepared using a jet polisher (TenuPol-5, Struers Inc., Cleveland, OH, USA) and perchloric acid solution at 15 V, 250 mA. The microstructural analysis was performed by a field-emission scanning electron microscope (FE-SEM, FEI Quanta 200F, FEI Company, Hillsboro, OR, USA) operated at an acceleration voltage of 15 kV, after which EBSD patterns were collected using a TSL OIM system (EDAX, Inc., Mahwah, NJ, USA) with a step size of 0.1 µm. Comparing the grain size and orientation between the surface and the center, as shown in Figure 15, dynamic recrystallization and growth of grains caused by stored work (or flow stress) are clearly observed in center region, with the increase in ram speed at the same die temperature. On other hands, the grain size in surface region gradually decreased with the increase in ram speed, which was probably originated from recrystallization and growth of sub-grains in peripheral coarse grains due to the increase in flow stress and frictional heat generated between the bearing and the workpiece, as predicted by the numerical analysis. 

## 4. Production Scale L-Shaped Sample Extrusion and Metallurgical Investigation of the White Line Defect

**Figure 16:**
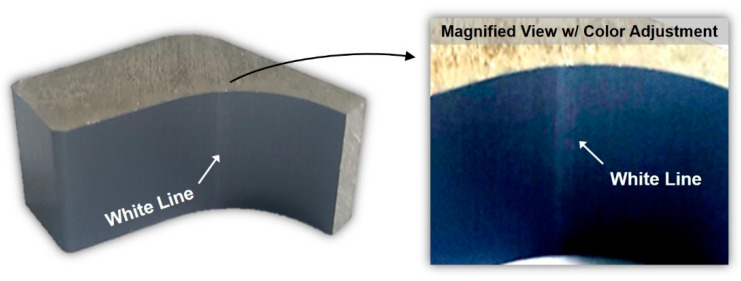
White line defect after anodizing treatment of the scaled-up L-shaped sample.

To investigate the potential application of the results of the laboratory experiment and numerical analysis in the production-scale manufacturing of L-shaped samples, we attempted to reproduce the surface defect observed in the laboratory-scale experiment in a production-scale L-shaped sample, which was around three times larger than the sample tested in the laboratory-scale experiment. Except for the ram speed and billet size, the extrusion conditions used for the scaled-up sample were almost exactly the same as those used in the laboratory-scale experiment. The ram speed for the production-scale extrusion process was determined based on the volumetric speed at the inlet and outlet. The scaled-up sample was subjected to anodizing treatment after extrusion to investigate the surface defects generated during manufacturing. Notably, the surface defect in the scaled-up sample was not observed immediately after the extrusion of the workpiece, but rather after the anodizing treatment process. The white line appeared on the surface of the inner curved region, the same location as that in which the surface defect was observed in the laboratory-scale experiment, with a surface gloss difference as shown in Figure 16. 

Thus, the location of white line defect generation could be anticipated prior to the product manufacturing process through a detailed numerical analysis in the perspective of the localized temperature and deformation, as depicted in Figure 11 and Figure 12, using advanced simulation techniques to provide practical information to optimize the process parameters, especially considering the lip shape and bearing dimensions that control the mass flow rate and temperature increase in the workpiece.

The white line defect on the surface of the workpiece can be observed either by the altered reflection of light owing to surface irregularities, or non-uniform anodizing during the aluminum coloring process performed after extrusion, which is closely related to the occurrence of the die line, grain boundary groove, and etching pit, and the disordered growth of the cellular structure during the grain etching step. Zhu et al. [30] reported that the altered reflection of light can be visibly detected beyond a surface roughness of 0.2 μm, based on the Rayleigh criterion of h>λ/8cosθ, which is also known as the smooth surface condition, considering a reflection angle of 60°. The parameters h, θ, and λ represent the height of the surface defect, the reflection angle, and the wavelength of the light, respectively. Wavelengths detectable by the human eye are typically around 0.38–0.78 μm.

**Figure 17:**
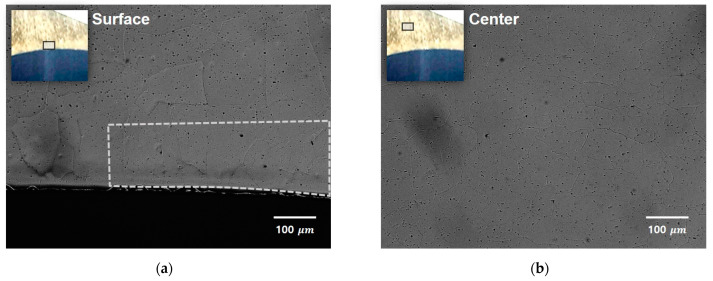
Microstructures of the defective region: (**a**) surface; and (**b**) center.

**Figure 18:**
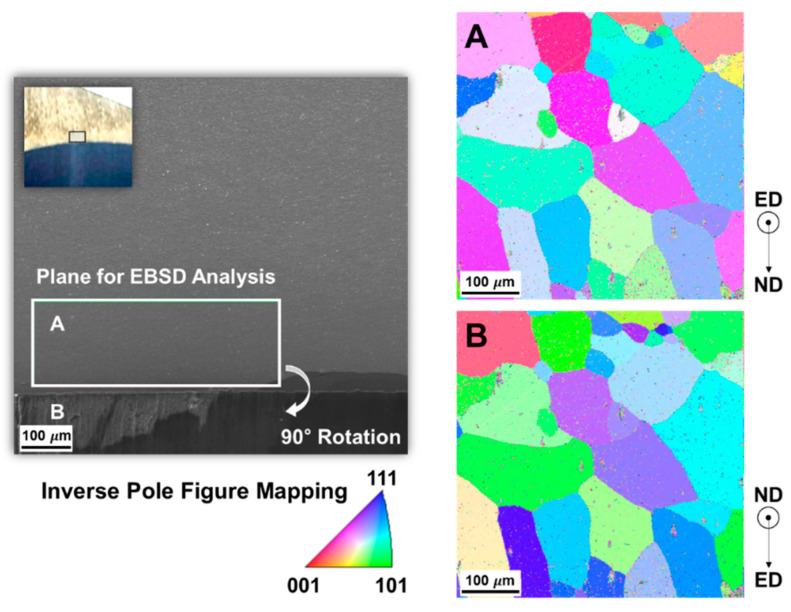
Inverse pole figure maps of the defective region: (**A**) parallel; and (**B**) perpendicular to the extrusion direction.

To comprehensively identify the white line defect generation mechanism, microstructural characterizations were performed on the as-received sample using EBSD and SEM. The EBSD maps were obtained at the location below the surface exhibiting the white line defect. No particular signs, such as the occurrence of coarse grains or the corrosion of grain boundaries, were observed by comparing the SEM images of locations both with and without the white line defect, as shown in Figure 17. The inverse pole figure (IPF) map in plane parallel to the extrusion direction shown in Figure 18 revealed that the grain orientations are relatively random on the observation plane, whereas they are similarly oriented along the <111> direction near the surface plane, particularly when the observation plane is rotated by 90°, such that it is perpendicular to the extrusion direction. The preferred orientation of the grains and locally densified grain boundary in the defect region probably causes non-uniform surface etching, thereby resulting in the disordered growth of the cellular structure during the formation of the anodic oxide film.

**Figure 19:**
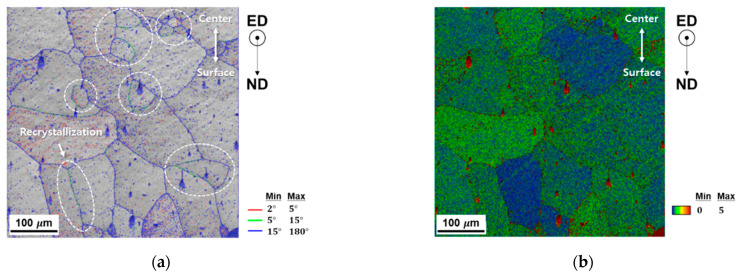
Distribution of: (**a**) the rotation angle; and (**b**) the kernel average misorientation of the defective region.

Rotation angle and kernel average misorientation (KAM) maps were obtained, as shown in Figure 19, to investigate the recrystallization due to the occurrence of high-density dislocation and estimate the distribution of the residual stress on individual measuring points to reveal preferable sites for grain corrosion. However, no severe deformation-induced grain boundary rotation or localized KAM traces between the surface and the center regions were detected. Furthermore, no pick-up mechanism was observed owing to inadequate homogenization of the billet. 

**Figure 20:**
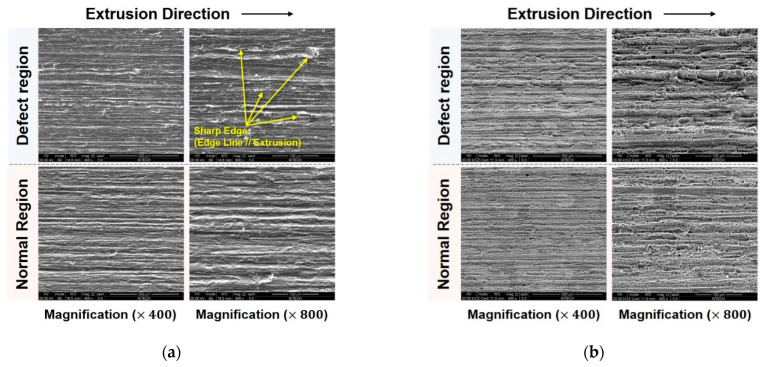
Surface roughness evaluation of the defective and normal regions: (**a**) before; and (**b**) after eliminating the anodic oxide layer.

In addition to the EBSD analysis, we attempted to evaluate the surface roughness of both the defect and defect-free regions of the sample using SEM images that were recorded before and after removing the anodic oxidation layer on the surface of the sample. Based on the width of the die line and the grain size, no surface irregularities related to the early oxidation on the grain boundaries and precipitates could be observed, as shown in Figure 20. 

The defective region had a rougher surface than the defect-free region, both with and without the anodic oxidation layer. The width of the die line and the grain size were 11.3 ± 4.1 μm and 42.6 ± 14.2 μm, respectively, in the defective region, and 8.4 ± 3.5 μm and 52.0 ± 19.4 μm, respectively, in the normal region. As the altered reflection of light and the difference in surface glossiness can be detected by the naked eye beyond a surface roughness of 0.2 μm, this quantitative measurement indicates that the defect can be attributed to either the variation in the surface roughness or the grain boundary groove that occurs due to the difference in the grain size between the defective and normal regions. This microstructural observation is also supported by the numerical and experimental observations of the scaled-down sample, as indicated by the friction concentration at the defect region owing to the rapid variation in the bearing length. Therefore, the temperature increase in the friction-dominated region locally accelerates not only the oxidation of the aluminum coating layer and the erosion of the nitride layer of the die, but also the fusion of aluminum oxide in the potential defect region along the extrusion direction. This defect mechanism possibly plays a significant role in the generation of surface irregularities, ultimately resulting in the occurrence of the visible white line in the final product after the surface treatment process. Therefore, the potential occurrence of this defect can be controlled by shortening the periodic maintenance duration of the die and re-adjusting the etching margin to successfully secure surface uniformity, besides the additional control of the process parameters and/or of the die and lip shape for extrusion.

## 5. Summary and Conclusions

In this study, we conducted a laboratory-scale experiment on the extrusion of an L-shaped sample at various ram speeds, considering the product yield rate required for industrial mass production, to obtain a comprehensive understanding of the potential mechanism of the white line defect generation. In an effort to figure out the severe changes in the physical state variable of the workpiece during the extrusion, an ALE technique-based numerical analysis was performed considering the strain rate- and temperature-dependent strain hardening of homogenized Al 6063 alloy. Accordingly, a combination of the Arrhenius-type equation and the Zener‒Hollomon parameter was used to obtain a reliable description and extrapolation of the flow stress response during severe deformations that involve substantial increases in the strain rate and temperature, which can be attributed to the thermal heat generated by intense sticking friction between the contact pairs and the plastic work of the workpiece. To investigate the underlying mechanism of the surface defects observed in the laboratory- and production-scale extrusion experiments, microstructural analyses based on SEM and EBSD were carried out at the defect location. In particular, the influence of strain and temperature localizations on the micromorphology and surface irregularity of the extruded sample, which would contribute to the altered reflection of light, resulting in visible detection of the white line, was investigated herein. Based on the numerical and experimental investigations presented, the following conclusions can be drawn:

1. Numerical extrusion analysis based on the ALE description predicted potential defect locations experiencing severe localization of deformation and temperature, as observed in the actual extrusion of L-shaped samples at both laboratory and production scales. 

2. The laboratory-scale experiment revealed that the surface defect generation has a strong dependence on the ram speed associated with the temperature localization on the failure region, which may be attributed to the increase in the sticking friction, especially at the location where the bearing length changes rapidly.

3. From the production-scale experiment, no coarse precipitates or grain boundary corrosion were observed in the metallurgical analyses, including in the deformation-induced boundary rotation and localized KAM traces, which indicated that the generation of Mg_2_Si precipitates during press quenching and aging treatments does not cause the white line defect. Furthermore, the evaluation of the width of the die line and the grain size revealed that surface irregularities were not generated due to the prior oxidation of the grain boundaries and precipitate. 

4. As the surface roughness of the defect region is relatively high compared to that of the defect-free region, both with and without the anodic oxidation layer, white line generation could be associated with streaking, which is one of the basic reasons for the altered reflection of light and the subsequent variation in the surface gloss of the defective region.

5. A potential cause of white line defect generation after anodizing treatment can be thus described as follows: the rapid increase in temperature owing to intense local friction not only accelerates the oxidation of the aluminum coating layer and the erosion of the nitride layer on the die bearing but also accelerates the fusion of aluminum oxide in the defective region along the extrusion direction. This defect mechanism can be discussed based on macroscopic and microscopic investigations, together with FE analyses, to further explain the occurrence of the visible white line defect after etching and anodizing treatment, along with the generation of a streak region, which would later be studied in more detail.

## Figures and Tables

**Table 1:** Evaluation of the physical quantities at each target condition of the compression tests.

Target Condition		Experimental Evaluation				
*T* [°C]	$\dot{\varepsilon }\;[s^{-1}]$	${\dot{\varepsilon }}_{\mathrm{avg}}\;[s^{-1}]$	*T*_avg_ [°C]	*T*_max_ [°C]	ε*_b_* [−]	*σ_y_* [MPa]
250	0.005	0.00385 (±0.00002)	250.50 (±0.68)	251.94 (±0.61)	0.5184 (±0.0013)	81.87 (±0.32)
	0.05	0.0383 (±0.0002)	248.25 (±0.57)	249.60 (±0.35)	0.4666 (±0.0258)	85.77 (±0.47)
	0.5	0.400 (±0.010)	254.07 (±0.79)	254.55 (±1.34)	0.4925 (±0.0130)	92.85 (±0.32)
	5	4.09 (±0.01)	253.27 (±0.98)	256.08 (±2.54)	0.4947 (±0.0076)	105.74 (±2.55)
350	0.005	0.00389 (±0.00001)	350.63 (±0.43)	351.84 (±0.90)	0.5027 (±0.0131)	21.30 (±0.56)
	0.05	0.0390 (±0.0002)	351.08 (±0.91)	357.96 (±3.33)	0.5087 (±0.0174)	37.20 (±2.28)
	0.5	0.401 (±0.000)	351.57 (±2.16)	353.30 (±1.77)	0.5085 (±0.0154)	51.89 (±0.66)
	5	4.20 (±0.02)	355.08 (±0.79)	355.18 (±3.85)	0.5301 (±0.0081)	65.05 (±0.28)
450	0.005	0.00393 (±0.00003)	451.08 (±0.88)	454.19 (±1.54)	0.5238 (±0.0156)	11.84 (±0.70)
	0.05	0.0392 (±0.0002)	447.50 (±0.47)	452.42 (±2.04)	0.4935 (±0.0045)	20.70 (±0.22)
	0.5	0.404 (±0.004)	446.04 (±2.00)	452.19 (±2.69)	0.5161 (±0.0197)	31.81 (±0.46)
	5	4.16 (±0.03)	452.31 (±1.53)	453.94 (±0.48)	0.5351 (±0.0017)	40.97 (±0.20)
550	0.005	0.00388 (±0.00010)	551.53 (±0.52)	551.82 (±0.72)	0.5062 (±0.0443)	6.73 (±0.60)
	0.05	0.0391 (±0.0007)	549.02 (±1.45)	550.81 (±0.52)	0.4885 (±0.0371)	11.70 (±0.96)
	0.5	0.402 (±0.001)	538.56 (±3.31)	551.33 (±3.01)	0.5092 (±0.0054)	18.43 (±0.25)
	5	4.05 (±0.03)	551.75 (±0.66)	552.39 (±0.89)	0.4819 (±0.0228)	26.05 (±0.19)

**Table 2:** Coefficients of the strain rate- and temperature-dependent strain hardening model.

Coefficients	Sub-Functions of the Arrhenius Type Equation			
	*α* [MPa^−1^]	*n* [−]	*Q* [KJ·mol^−1^]	ln*A* [s^−1^]
*c*_0_	0.0408	5.6149	205.3456	31.5017
*c*_1_	−0.0349	11.9208	1374.7045	221.5863
*c*_2_	0.2327	−89.5042	−8253.6344	−1346.7027
*c*_3_	−1.0630	272.3083	23,605.6538	3928.3140
*c*_4_	2.1408	−433.1257	−35,110.4100	−5953.9828
*c*_5_	−1.6026	288.8021	21,304.3244	3669.3198

**Table 3:** Information related to the numerical simulation of the L-shaped sample extrusion process.

Initial Temperature, *T* [°C]			Thermal Expansion Coefficient, *α* [10^−6^ m/(m·°C)]	Thermal Conductivity, *κ* [W/(m·K)]	Convective Heat Transfer Coefficient, *h* [W/(m^2^·K)]	Ram Speed, *ν* [mm·s^−1^]	Shear Friction Factor, *m* [−]
Billet	Die/lip	Container					
430	414	430	22	180.19	5	3.5, 6, 9, 12	0.8

## Data Availability

The data presented in this study are available on request from the corresponding author.

## Appendix A. Arbitrary Lagrangian–Eulerian Description

The numerical foundation of the ALE method is based on the principle of virtual work:

(A1) $$\int_{V}\sigma_{ij}\delta e_{ij}dV=\int_{V}f_{i}^{B}\delta u_{i}dV+\int_{S}f_{i}^{S}\delta u_{i}dS,\;$$

where $\sigma_{ij}$, $e_{ij}$, $f_{i}^{B}$, $f_{i}^{S}$, V, S, and $\delta u_{i}$ denote the Cauchy stress and deformation tensors, body force per unit volume, externally applied surface traction, volume and surface of the deformed body on which the tractions are applied, and virtual displacement, respectively. Considering the computational reference domain at time t and the time derivative of Equation (A1), the computational nodal points can be held constant to yield the numerical formulation of the ALE method [31]:

(A2) $$\underset{V}{\int }\frac{\partial \delta u_{i}}{\partial x_{j}}\left[\left(-\frac{\partial {\hat{v}}_{j}}{\partial x_{p}}\right)\sigma_{ip}+{\dot{\sigma }}_{ij}^{J}+\frac{\sigma_{ip}}{2}\left(\frac{\partial v_{j}}{\partial x_{p}}-\frac{\partial v_{p}}{\partial x_{j}}\right)+\frac{\sigma_{jp}}{2}\left(\frac{\partial v_{i}}{\partial x_{p}}-\frac{\partial v_{p}}{\partial x_{i}}\right)\right]dV\;+\underset{V}{\int }\frac{\partial \delta u_{i}}{\partial x_{j}}\left[\left({\hat{v}}_{p}-v_{p}\right)\frac{\partial \sigma_{ij}}{\partial x_{p}}+\sigma_{ij}\frac{\partial {\hat{v}}_{p}}{\partial x_{p}}\right]dV-\underset{V}{\int }\delta u_{i}\left[\left({\hat{v}}_{p}-v_{p}\right)\frac{\partial f_{i}^{B}}{\partial x_{p}}+f_{i}^{B}\frac{\partial {\hat{v}}_{p}}{\partial x_{p}}\right]dV,\;-\underset{V}{\int }\delta u_{i}\left[\left({\hat{v}}_{p}-v_{p}\right)\frac{\partial \sigma_{ij}}{\partial x_{p}}+\sigma_{ij}\frac{\partial {\hat{v}}_{p}\left(x_{j}=0\right)}{\partial x_{p}}\right]n_{j}dS=\underset{V}{\int }\delta u_{i}{\dot{f}}_{i}^{B}dV+\underset{V}{\int }\delta u_{i}{\dot{f}}_{i}^{S}dS$$

where $v_{i}$, ${\hat{v}}_{i}$, ${\dot{\sigma }}_{ij}^{J}$, and ${\dot{f}}_{i}$ denote the material and mesh velocities, the Jaumann rate of Cauchy stress, and the material rate of change of the force, respectively. Equation (A2) is regarded as a complete and general formulation that covers all the aforementioned numerical techniques and can describe various types of finite deformation problems with generalized loading and boundary conditions. As ${\hat{v}}_{i}$ is arbitrary, the ALE formulation generally has no intrinsic dependence on the mesh. If the material velocity is equal to the mesh velocity, i.e., $v_{i}={\hat{v}}_{i}$, Equation (A2) reduces to the UL formulation; if the material velocity is set to zero, i.e., ${\hat{v}}_{i}=0$, it reduces to the Eulerian formulation.

In the standard finite element procedure based on numerical discretization, Equation (A2) can be expressed on an elemental level as follows:

(A3) $$k_{ij}v_{j}+{\hat{k}}_{ij}{\hat{v}}_{j}=f_{i},$$

where $k_{ij}$, ${\hat{k}}_{ij}$, and *f_i_* denote the stiffness components related to the material and mesh velocities, and the external loading at the nodal points, respectively. Considering the description of the relative velocity field between the material and the mesh, supplementary conditions can be devised with a transfinite mapping that defines a relation between the material and mesh motions, resulting in a non-zero value of the Jacobian of the mapping function between the material and the mesh:

(A4) $${\hat{v}}_{j}=a_{j}+b_{\left(j\right)}v_{\left(j\right)}.$$

Here, there is no summation on j. The characteristic of the ALE method can be preserved in the relative velocity between the material and the mesh with the choice of the coefficients *a* and *b*: for the Lagrangian formulation, $a_{j}=0$, $b_{\left(j\right)}=1$; for the Eulerian formulation, $a_{j}=b_{\left(j\right)}=0$. By modifying the element stiffness to aid the defined relative velocity, the number of unknows to be solved can be reduced to be the same as that in the traditional FE computation:

(A5) $$\left[k_{ij}^{\left(m\right)}+k_{ij}^{\left(g\right)}b_{j}\right]v_{j}=f_{i}-k_{ij}^{\left(g\right)}a_{j},$$

or equivalently,

(A6) $$K_{ij}v_{j}=F_{i}.$$

The existence of the one-to-one mapping between the material and mesh domains can be satisfied only if the boundaries of each domain coincide with each other, i.e., $\left(v-\hat{v}\right)\cdot n=0$, where n is the normal vector at any given point on the specified boundary. Physically, this implies that no normal convective velocity is allowed across the boundary if the material points remain on the surface.

After computing the material velocity at each nodal point, mesh smoothing is conducted during the mesh refinement stage of the Eulerian process in the ALE method. This preserves the topology of the initial mesh unlike the classical remeshing techniques applied in the UL method. After the Lagrangian process, each node is relocated based on a volume-smoothing algorithm that computes the weighted average of the element centers of the elements surrounding the node:

(A7) $$x_{n}^{i+1}=\sum_{e=1}^{n_{s}}V_{e}^{i}x_{e}^{i}/\sum_{e=1}^{n_{s}}V_{e}^{i},$$

with

(A8) $$x_{e}^{i}=\frac{1}{n_{e}}\sum_{k-1}^{n_{e}}x_{k}^{i},\;$$

where $x_{n}^{i+1}$, $x_{e}^{i}$, $x_{k}^{i}$, and $V_{e}^{i}$ denote the position vectors of a node n, the eth element center, the kth node of the eth element, and the volume of the surrounding eth element, respectively; $n_{s}$ and $n_{e}$ are the node number of the eth element and the number of surrounding elements, respectively. After the mesh refinement process, the state variables, including the stress, strain, and temperature, are updated at each integration point on the newly defined mesh configuration obtained from the previous mesh, while guaranteeing the conservation of the state variables during mesh motion. To map the state variables between the material and mesh (reference) domains of X and χ, the material derivative of any scalar function f(x,t) is expressed in terms of the reference time derivative and the spatial derivate:

(A9) $${\frac{\partial f}{\partial t}|}_{X}={\frac{\partial f}{\partial t}|}_{\chi }+\left(v_{i}-{\hat{v}}_{i}\right)\frac{\partial f}{\partial x_{i}}={\frac{\partial f}{\partial t}|}_{\chi }+c_{i}\frac{\partial f}{\partial x_{i}},$$

or, equivalently,

(A10) $$\Delta f{|}_{X}=\Delta f{|}_{\chi }+\Delta t\left(v_{i}-{\hat{v}}_{i}\right)\frac{\partial f}{\partial x_{i}},$$

where x and $c_{i}$ denote the spatial domain and the convective velocity that represents the relative velocity of the material with respect to the mesh in the laboratory system, respectively. An incremental analysis based on the material derivative enables the tracking of the material deformation and the corresponding state variables in the space‒time integration of the ALE description.
